# The impact of health promotion on trachoma knowledge, attitudes and practice (KAP) of staff in three work settings in remote Indigenous communities in the Northern Territory

**DOI:** 10.1371/journal.pntd.0005503

**Published:** 2017-05-24

**Authors:** Fiona D. Lange, Kelly Jones, Rebecca Ritte, Haley E. Brown, Hugh R. Taylor

**Affiliations:** 1 Indigenous Eye Health, Indigenous Health Equity Unit, Centre for Health Equity, Melbourne School of Population and Global Health, The University of Melbourne, Victoria, Australia; 2 Indigenous Health Equity Unit, Centre for Health Equity, Melbourne School of Population and Global Health, The University of Melbourne, Victoria, Australia; 3 Melbourne School of Population and Global Health, The University of Melbourne, Victoria, Australia; RTI International, UNITED REPUBLIC OF TANZANIA

## Abstract

**Background:**

Globally, trachoma is the leading cause of infectious blindness and Australia is the only developed country with endemic trachoma. It is found in remote Indigenous communities burdened with poverty, overcrowding and poor hygiene. Lack of culturally appropriate health promotion, a small trachoma workforce and lack of awareness and support for trachoma elimination in general, were early barriers.

**Methods:**

A cross-sectional pre-post study using a convenience sample, was conducted in clinics, schools and community work-settings from 63 of the 82 remote Aboriginal communities identified as being at risk of trachoma in the Northern Territory (NT). The study assessed the effect of a multi-component health promotion strategy aimed at increasing knowledge, attitude and practice amongst health, education and community support settings staff. Data were collected between 2010 and 2012. The health promotion initiatives were introduced in communities in staggered delivery over a one-year period; 272 participants were surveyed at baseline and 261 at follow-up.

**Results:**

Trachoma related knowledge, attitudes and practice increased across all settings and for all primary outcome measures. Across all settings, there was a significant increase in the proportion of participants reporting the most important thing to do if a child has a ‘dirty’ face is to ‘wash it every time its dirty’ (61.6% *cf* 69.7%; *X*^2^p = 0.047), a significant reduction in the proportion of respondents answering ‘no’ to the question “Is it normal for kids to have dirty faces in your community’ (40.5% *cf* 29.6%; *X*^2^p = 0.009) and a significant increase in reported capacity to teach others about trachoma prevention (70.8% *cf* 83.3%; *X*^2^p <0.001).

**Conclusion:**

Health promotion was associated with increased trachoma knowledge, attitude and practice amongst health, education and community support staff working with children and in remote NT communities. In the early stages of the trachoma health promotion program, this increased trachoma awareness and improved local workforce capacity and support for trachoma elimination in three health promotion settings in remote communities in the NT.

## Author summary

Australia is the only high-income nation with endemic trachoma. It is prevalent in remote Indigenous communities, although it was eliminated from wider society in Australia over one hundred years ago. Trachoma elimination across vast areas of remote Australia is an enormous challenge. Community members are highly mobile, English may be the second, third or fourth language in multiple Indigenous language groups, and trachoma is merely one of many complex health and social issues. A small number of trachoma program staff and frequent staff turnover in all work settings also limit progress. Trachoma elimination has focused on the Surgery & Antibiotic elements of the SAFE strategy. However, Facial cleanliness & Environmental improvements are critical for sustained progress towards elimination. Health promotion offers multiple, engaging approaches to help increase awareness that trachoma is simple to treat and that clean faces and improved hygiene practices help children be healthy and strong. In addition, safe and working bathroom (washing) facilities are needed to carry out hygiene practices. The two target groups for trachoma health promotion were staff who work in remote communities, and children and their carers (mothers, aunties, grandmothers, or other family members). The participants in this study, in the early stages of the health promotion program provides evidence that health promotion made a contribution to improved trachoma knowledge, attitudes and to a lesser extent practices in staff who provided education and support to children and families in trachoma endemic communities. This was achieved in a relatively short time despite high levels of staff turnover. Health promotion for hygiene related behaviour change requires multiple-sector support crucial to operationalise clean faces/hygiene practices more widely and to provide safe and working bathroom facilities in homes, schools, clinics and community work settings. This paper adds to the literature by illustrating some success achieved in a trachoma health promotion strategy in the Northern Territory and may support trachoma elimination in Australia and globally.

## Introduction

Trachoma is the world’s leading cause of infectious blindness. Globally, trachoma is linked to poverty, household crowding, limited access to water and poor hygiene [1,2]. Active infection with *Chlamydia trachomatis* is usually found in young children and transmitted by infected eye and nose secretions. A child may have between 30 and 40 episodes of reinfection during their childhood and up to 160 to 180 infections may occur in total. The resultant scarring and in-turned eyelashes (trichiasis) damage the cornea leading to blindness in older people [3].

Australia is the only high-income country with endemic trachoma, one of four eye conditions leading to vision loss and blindness in Indigenous Australians [4,5]. Trachoma disappeared from most of Australia in the early 1900s when water and sanitation infrastructure improved living conditions [6]. Yet it persists in remote Aboriginal communities where limited and overcrowded housing, lack of working bathrooms and poor hygiene, are still common [7,8,9]. Additionally, children with persistent eye and nose secretions sometimes go unnoticed and is not always commonplace to wash a child’s face whenever it is dirty [10,11].

Australia is committed to the “Alliance for Global Elimination of Trachoma by 2020” utilising the World Health Organization’s SAFE strategy to eliminate trachoma. The four elements are; Surgery for trichiasis (S), Antibiotics for active trachoma infection (A), Facial cleanliness to reduce transmission (F) and Environmental improvements (E) [12,13].

The association between facial cleanliness, nasal discharge and trachoma is well established [14,15,16,17]. In Australia, the prevalence of clean faces (without nasal or ocular discharge) is a mandatory reporting indicator for trachoma surveillance and promoting facial cleanliness is central to the public health management of trachoma [18]. ‘Clean face, strong eyes’ is the key message in trachoma health promotion [19].

Government programs increased in 2009 providing coordinated, visiting screening and antibiotic treatment programs in communities at risk of trachoma. Trichiasis screening and referral for surgery are provided by staff in remote community clinics [18]. Identified barriers to implementing the SAFE strategy include a lack of culturally appropriate resources and that trachoma was not considered a priority by staff in remote clinic work settings [20]. Multiple component trachoma resources, based on the SAFE strategy, were developed for working with individuals and for use at the population level. In the early stages of the program reported in this study, resources were used directly with staff members to develop and support a workforce for trachoma elimination within remote communities. The health promotion strategy used the established settings-based health promotion approach [21]. The goal of working with local staff was to increase their trachoma related knowledge, attitudes and practice making it possible for them to become intermediaries and agents of change with the community population.

The expanded trachoma screening and control activities 2010 resulted in trachoma rates declining considerably from 14% in 2009 (1–9 yrs.) to 4% in 2012 (5-9yrs) in the NT. However, of the 82 communities considered at risk in the NT in 2010, 28% remained with endemic trachoma and 37% were considered hyper-endemic in 2012 [22]. This study examines changes in the trachoma-related knowledge, attitudes and practice of staff in clinic, schools and community work settings in 63 of the 82 communities at risk of trachoma in the NT, following exposure to the new health promotion initiatives.

## Methods

### Ethics statement

Ethics approval was provided by the University of Melbourne Human Research Ethics Committee (0932512.1). Participants were given an information sheet detailing the study, the voluntary nature of participation and participant consent was implied through the completion of the questionnaire. RESTRICTIONS: Ethics approval for the study required that personal information (names) and geographic (community name) location must be omitted. Data storage, security and access adheres to the University of Melbourne Policy on the Management of Research Data and Records. LINK: http://records.unimelb.edu.au/ Raw data for this research cannot be made publicly available for ethical reasons, as it would breach compliance with the protocol approved by the University of Melbourne Human Research Ethics Committee.

### Study design

A pre-post, cross-sectional study was used to evaluate new health promotion strategies implemented between 2011 and 2012 in 3 different work settings (health clinics, schools and community work-places). A one-page survey was designed in plain English for ease of understanding and participation in busy work places. Self-complete baseline surveys measuring knowledge, attitude and practice (KAP) were conducted between August 2010 and June 2011 in the three settings prior to introducing the health promotion intervention across the NT. Follow-up surveys were identical but included 3 questions about the resources and images as prompts. These were conducted from February-July 2012 to determine changes in trachoma KAP amongst staff in the three work settings.

### Health promotion settings

The settings-based approach integrates trachoma health promotion program delivery with local programs and priority issues and approaches with the aim of cultivating support in clinics, schools and community support settings. Multiple settings and simultaneous health promotion approaches are commonly used internationally and in Australia to increase effectiveness and promote synergistic outcomes [23,24,25,26]. At the time of the study there was one trachoma educator for the 86 communities at risk in the NT. Trachoma nurses conducted screening and treatment and some health education in the communities once or twice a year. A local workforce to support trachoma and hygiene education was needed to educate and support remote community adults and children in eliminating trachoma. A number of Indigenous community members were employed in these settings and many Indigenous community members engaged with the clinic, school or community activity centres on a daily basis. Staff were considered important intermediaries who could teach children and deliver health messages to children’s carers about trachoma elimination and could identify children in need of help. Due to the high turnover of staff in all workplaces in remote Indigenous communities, the program aimed to implement trachoma KAP into existing systems and workplace practices, to induct new staff and inculcate the information into the corporate knowledge within settings and beyond the tenure of staff members.

### Health promotion resources and initiatives

Health promotion initiatives were based on the ‘Trachoma Story Kit’ developed in response to lack of culturally appropriate material for Australia. Input came from Aboriginal Health Services, Departments of Health and Education, NGOs, community programs and environmental health. The Ngumbin Reference Group of Elders and Aboriginal Health Workers from the Katherine West region of the NT advised throughout the development phase on cultural safety and acceptability of the resources^.^ They recommended the resources be placed in clinics, schools and community work-places because they viewed *trachoma elimination as everyone’s business* [27]. All resources featured the program mascot—Milpa (eye in Warlpiri language) the Trachoma Goanna and the slogans *"Clean Faces*, *Strong Eyes" and “Wash your face whenever its dirty”* to support new social norms for facial cleanliness. Clinic materials included clinical training tools and patient education material. Additionally, an e-learning module—based on the Trachoma Story Kit—was developed by Remote Area Health Corps and Indigenous Eye Health for urban health practitioners doing short-term contracts in remote Indigenous communities [28]. Schools are a key setting for trachoma education globally, and as children can be change agents for trachoma elimination [29,30]. lesson plans were developed in line with the school curriculum [19]. Community workplace materials featured realistic images, stories and multi-media to engage family support services. Population level initiatives included public football events [31] and television and radio adverts reinforced the key messages with Milpa the Trachoma Goanna. The multi-media strategies maximised program reach through the wide footprint of digital media channels in remote Indigenous communities across the NT and the multiple health promotion activities were designed for a complementary and synergistic effect. Table 1, shows the suite of health promotion initiatives that make up ‘the intervention’. Participants were asked if they had seen or heard one or more activity. Prior to the intervention, no known population-based trachoma health promotion had been introduced across the NT.

**Table 1:** Health promotion initiatives and delivery timelines.

Health Promotion Initiative	Reach	Timeline
Trachoma Story Kits	626 kits	August 2010 to June 2012
Trachoma E Module based on Trachoma Story Kit*	109 modules completed	Jan 2011 to July 2012
Trachoma health promotion at Australian Football League games#	2 games	July 2011 and July 2012
Trachoma health promotion community performance	30 live performances	July 2011 to July 2012
Childhood setting safety mirrors installed	50 work settings	Aug 2010 to Dec 2011
Trachoma health promotion football clinics	6 football clinics	July 2011 to July 2012
Trachoma health promotion posters	3000 posters	Feb 2011 to July 2012
Trachoma health promotion advert–TV^##^	1008 community service announcements	July 2011 to Feb 2012
Trachoma health promotion advert–Radio	3840 community service announcements	Jan to July 2012

* Remote Area Health Corps clinical e-training module

^#^ Approximately 10,000 spectators at each game

^##^ Footprint and reach for the broadcasts of 75,000 per month during 6 months of transmission

### Study participants

Study participants came from 63 of 82 remote communities in the Northern Territory identified ‘at risk of trachoma’ by the National Trachoma Surveillance and Reporting Unit. Reported population size in communities ranged from 30 to 3000 participants. Participation in the study was voluntary. Participants in the study were given an information sheet detailing the study, the voluntary nature of participation and participant consent was implied through the completion of the questionnaire. All staff in each work setting were invited to participate at the time of the baseline and the follow-up surveys. This was conducted either at their place of work or in bigger regional centres during annual work place training. Due to the frequent staff turnover in clinics, schools and community service work places, it was expected that staff in the follow up surveys would not necessarily be the same as in the baseline survey. The pre/post participants were not matched in this study. Opportunistic recruitment of survey participants was conducted in person by two researchers from The University of Melbourne, a Trachoma Educator, four nurses and one clinic co-coordinator from Centre for Disease Control in the NT, the Katherine West Health Board (KWHB) trachoma co-ordinator and seven KWHB clinic managers. Translation of the questionnaire was provided as needed. Personal identifying data were not recorded. There was a mix of Indigenous and non-Indigenous staff in all settings. Health clinic participants were Aboriginal Health Practitioners, doctors, nurses and other allied health practitioners. A number of ancillary staff in clinics also participated, including local community members working as receptionists, liaison officers or drivers. Participants from the school/pre-school setting included teachers, teaching assistants and tutors including Indigenous community members. Community support work-settings were child care centres, family wellbeing centres, sport and recreation programs that employed childcare staff and assistants, family support staff, sport and recreation and youth workers. Calculations of sample size were performed using Openepi software. A minimum of 150 participants for both baseline and follow up was required to detect a 15% change in the proportion of participants reporting acceptability of clean faces amongst children aged 0–9 from 40% to 25% at the significance criterion of 0.05 and a power of 0.80.

### Data collection and measures

Questionnaire items were developed and piloted with community members as previously reported [9]. The plain English self-administered 17-item questionnaire comprising both closed and open-ended questions took approximately 10 minutes to complete. Ten questions related to knowledge, three to attitudes and four to practice. Clinic staff were asked to complete an additional three questions relating to trachoma and screening. Data items relating to ‘clean face’ acceptability and knowledge around preventive actions used the WHO ‘clean face’ definition and the commensurate community nomenclature for referencing ‘dirty’ faces was used. This terminology was supported through pilot testing of the questions with the Ngumbin Reference Group (NRG) [10].

### Statistical analysis

Data was analysed using IBM SPSS v.13. Baseline measures of knowledge, attitude and practice were summarised using descriptive statistics and data was treated as two independent, cross-sectional samples. The three main outcome measures are reported as the proportion difference between baseline and follow-up and significance was tested using the Chi-Square test for proportional differences and AVOVA for testing independent associations. Data were stratified by workplace setting. Crude associations between important trachoma prevention outcomes (knowledge about the spread of trachoma, the ability to teach, most important thing to do and who to treat, attitude towards ‘dirty’ faces and treatment) were assessed across settings using unconditional logistic regression with clinics being the reference category. To assess whether there was a significant change across the pre- and post-intervention time period by setting, an interaction term for pre- and post- intervention was included in the logistic regression model and significant interaction of the pre/post period by work setting was assessed using the Wald statistic. All tests of significance were two-sided and p value of <0.05 was considered significant. The primary outcomes of interest were significant differences between baseline (pre-) and follow-up (post-) in the proportion of responses and effect of the intervention on these outcomes. The primary outcome measure for knowledge used was ‘*What is the most important thing to do if a child has a dirty face*?*’*, dichotomized as ‘wash face whenever its dirty/wipe clean with a tissue’ and ‘wash each morning/wash morning and night/unsure’. The primary attitude based measure was ‘*It is normal in your community for children to have dirty faces’* dichotomized as ‘Yes’ or ‘No/Unsure’ and the primary practice outcome measure used was *‘I am able to teach others about trachoma prevention’* and was also dichotomized as ‘Yes’ or ‘No/Unsure’. It should be noted that this question was variously answered as being ‘acceptable or ‘usual’. All missing data were excluded from analysis (generally < 5%).

## Results

Table 2 shows the distribution of correct questions by 272 participants pre- and 261 participants post-intervention in three work place settings and for the group as a whole. The majority of pre- and post- participants were from clinics n = 272(73.5%) and n = 148 (56.7%) respectively. The response from schools and community work places was much lower but increased in the post-intervention survey after the introduction of resources kits and other health promotion in their area. Most participants (94.8%) from the three settings overall could correctly define trachoma and knew it could lead to blindness (96.1%), yet one in five (17.9%) did not know they lived and worked in a trachoma endemic area. Over four in five (84.6%) reported that trachoma could be spread from person to person (the main transmission route) yet only 70.5% understood that contamination by towels or bedding is possible.

**Table 2:** Distributions of correct questions by work setting: Pre- and post- intervention.

Question	Response	All Pre- % (n = 272)	All Post- % (n = 261)	Clinic Pre- % (n = 200)	Clinic Post- % (n = 148)	School Pre- % (n = 28)	School Post- % (n = 58)	Community Pre- % (n = 44)	Community Post % (n = 55)
What is trachoma?	Correct definition	91.0 (222)	94.8 (235)	**90.8 (168)**	**96.4**^**c**^ **(134)**	82.6 (19)	96.4 (53)	97.2 (35)	88.9 (48)
Is trachoma in your community?	Yes	78.8 (208)	82.1 (211)	79.9 (155)	82.4 (122)	63.0 (17)	75.0 (42)	83.7 (36)	88.7 (47)
Can trachoma be spread from person to person?	Yes	84.6 (230)	88.8 (229)	90.0 (180)	94.6 (140)	60.7 (17)	77.2 (44)	75.0 (33)	84.9 (45)
Can people get trachoma from shared bedding and towels?	Yes	70.2 (184)	70.5 (179)	73.3 (140)	71.5 (103)	57.1 (16)	64.3 (36)	65.1 (28)	74.1 (40)
Can trachoma lead to blindness?	Yes	94.5 (257)	96.1 (249)	97.0 (194)	98.6 (146)	82.1 (23)	91.2 (52)	90.9 (40)	94.4 (51)
Is trachoma simple to treat?	Yes	**85.2 (230)**	94.4* (243)	**89.5 (179)**	**97.3**^**d**^ **(144)**	**63.0 (17)**	**86.0**^**i**^ **(49)**	**79.1 (34)**	**94.3**^**j**^ **(50)**
Would you know if someone has trachoma?	No	46.9 (130)	48.4 (124)	**46.1 (83)**	**31.1**^**e**^ **(46)**	31.3 (21)	68.7 (46)	44.8 (26)	55.2 (32)
If a child has a dirty face, what is the most important thing to do?	Wash face whenever its dirty	**61.6 (167)**	**69.7**^**a**^ **(182)**	62.5 (125)	68.9 (102)	64.3 (18)	69.9 (40)	55.8 (24)	72.7 (40)
If trachoma is found in a child, who has to take trachoma medicine?	Everyone who sleeps in same house (child and contacts)	71.0 193)	73.2 (191)	76.0 (152)	81.1 (120)	57.1 (16)	60.3 (35)	56.8 (25)	65.5 (36)
Is it normal in your community for children to have dirty faces?	Yes NO	**40.5 (104)**	**29.6**^**b**^ **(71)**	**41.6 (79)**	**25.7**^**f**^ **(36)**	32.1 (9)	38.5 (20)	41.0 (16)	31.3 (15)
Normal for old people to have poor vision /sick eyes	Yes NO	70.7 (180)	77.0 (184)	**71.4 (135)**	**85.1**^**g**^ **(120)**	67.9 (19)	62.0 (31)	69.2 (27)	68.8 (33)
I feel comfortable talking about hygiene issues with others	Yes	90.5 (237)	92.0 (230)	91.8 (178)	93.1 (134)	78.6 (22)	92.5 (49)	92.5 (37)	88.7 (47)
I am able to teach others about trachoma prevention	Yes	**70.8 (184)**	83.3* (209)	**76.0 (146)**	**88.4**^**h**^ **(129)**	**50.0 (14)**	**73.1**^**j**^ **(38)**	**60.0 (24)**	**79.2**^**l**^ **(42)**
List three things you can do to stop trachoma transmission	3 correct responses	88.7 (133)	97.6 (244)	88.8 (95)	99.3 (145)	90.0 (18)	96.3 (52)	87.0 (20)	94.0 (47)
I know enough about trachoma	Yes			**35.2 (63)**	62.7* (84)	na	na	na	na
I can flip eyelids to check for trachoma	Yes			70.3 (128)	75.4 (104)	na	na	na	na
I know how to find trichiasis	Yes			**50.8 (91)**	73.7* (98)	na	na	na	na
Reported seeing 1^+^ intervention**	Yes		69.2 (162)		72.3 (99)		50.0 (22)		77.4 (41)

X^2^ p = ^a^0.047; ^b^0.009; ^c^0.04; ^d^0.005; ^e^0.006; ^f^0.001; ^g^0.002; ^h^0.004; ^i^0.02; ^j^0.04; ^k^0.02; ^l^0.04; *<0.001.

### Overall

Significant differences were found for all three main outcomes of interest post-intervention. The overall proportion of participants reporting the key health promotion message (the most important thing to do with a child who has a dirty face was to ‘*wash the face whenever its dirty’*) increased significantly post-intervention (61.6% *cf* 69.7%; *X*^2^p = 0.047). Participants reporting it was ‘*normal for children to have a dirty face*’ decreased significantly (40.5% *cf* 29.6%; *X*^2^p = 0.009) and those reporting being able to teach others about trachoma prevention increased significantly (70.8% *cf* 83.3%; *X*^2^p<0.001). Additionally, participants reporting an understanding that trachoma was simple to treat increased overall (85.2% *cf* 94.2%; *X*^2^p<0.001). When asked about seeing/hearing one or more of the suite of health promotion initiatives which made up the health promotion intervention, 69.2% had seen/heard at least one.

### Clinics

When stratified by setting, participants from health clinics reported the greatest change in trachoma knowledge, attitude and practice. There was a significant increase in the proportion of participants reporting they knew at least two correct ways to stop trachoma transmission increasing from 88.8% to 99.3% (*X*^2^p<0.001). There was a statistically significant change in the proportion of respondents answering 'no' to the question “Is it normal for kids to have dirty faces in your community”? (41.6% *cf* 31.3%; *X*^2^p = 0.001). Clinic staff reporting they knew enough about trachoma increased post-intervention (35.2% *cf* 62.7%; *X*^2^p = 0.004) as did the proportion of participants reporting the correct definition of trachoma (90.8% *cf* 96.4%; *X*^2^p = 0.04). Although those reporting being able to screen for active trachoma only increased marginally (70.3% *cf* 75.4%; *X*^2^p = 0.32) post-intervention, knowledge about how to identify trichiasis increased significantly (from 50.8% to 73.7%; *X*^2^p<0.001). When asked post-intervention about seeing/hearing one or more of the suite of health promotion initiatives which made up the trachoma prevention intervention, 72.3% reported they had seen/heard at least one.

### Schools

School staff showed a significant increase post-intervention in the proportion of participants reporting that trachoma was simple to treat from 63.0% to 86.0% (*X*^2^p = 0.02). There was an indication of an increase in the proportion of participants reporting feeling comfortable talking about hygiene issues with others, post- intervention (78.6% *cf* 92.5%; *X*^2^p = 0.07). There was a significant increase in the proportion of participants reporting they are able to teach others about trachoma prevention (50.0% *cf* 73.1%; *X*^2^p = 0.04). Only 50.0% of school staff reported they had seen/heard at least one of the trachoma health promotion interventions.

### Community workplace

Participants from the community work-settings showed an indication of an increase in the proportion of participants reporting that the most important thing to do if a child has a dirty face is to wash it whenever its dirty (55.8% *cf* 72.7%; *X*^2^p = 0.47) however this was not significant. The proportion of participants reporting trachoma was simple to treat (79.1% *cf* 94.3%; *X*^2^p = 0.02) and reporting feeling able to teach others about trachoma prevention (60.0% *cf* 79.2%; *X*^2^p = 0.04) post intervention was higher post-intervention. Of community work setting staff, 77.4% reported they had seen/heard at least one, the highest of all the work-settings.

## Discussion

In this study, the trachoma related KAP of staff who provided education and support to families and children was assessed during the early stages of health promotion in trachoma endemic communities. The multi-faceted interventions for trachoma health promotion were established in the settings where people engaged in daily activity, with the aim of attaining synergistic effects and sustained population-wide behaviour change. There is renewed interest in the settings-based approach to facilitate health promotion and public health action [21] and it was anticipated that embedding trachoma related KAP in work settings may redress a previous finding that trachoma was not considered a priority in remote health services [20]. Trachoma elimination in vast areas of remote Australia is an enormous challenge and the workforce for trachoma elimination in the NT is small. During this study, visiting trachoma nurses conducted annual screening and treatment and provided education sessions mainly with children in schools and with local staff in clinics and community work places. It was anticipated, local staff could support trachoma elimination as intermediaries with families to support trachoma elimination, an approach used with an Indigenous children’s ear-health program [32]. Local staff have a unique capacity to engage with resources, facilitate interaction and stimulate discussion with children and carers in an everyday, supported context [33], such as exists in remote work places. Importantly they can encourage supportive practices and encourage behaviour change for children and families. However, while the KAP of staff will generally improve after health education in most workplaces, a significant limitation in remote communities is the considerable level of staff turnover. In clinics about 60% of staff change annually [34] and in schools, staff retention is commonly measured in months rather than years [35]. Embedding trachoma related KAP into workplace practices was a priority to establish and operationalise trachoma elimination in supportive work places, beyond staff tenure.

Underlying the ill health and health related behaviours of Indigenous Australians are many historical, geographical and socioeconomic determinants [36,37,38,39]. In addition, past assimilation policies included the forcible removals of Indigenous children from their families often occurring with allegations of child neglect and poor hygiene [40,41]. Subsequently, addressing hygiene related health is complex. A key barrier to eliminating trachoma and addressing other hygiene-related health issues, is normalisation of dirty faces in young children in some remote Indigenous communities [42,43]. Indigenous and non-Indigenous staff and community members do not always notice children’s dirty faces or help to clean eye or nose secretions from the faces of small children [10]. Non-Indigenous staff may be hesitant to assist or discuss clean faces for fear of shaming, being rude or intrusive, or they may simply not know how best to help. A direct, non-blaming, practical approach is crucial when addressing hygiene/health issues with Indigenous children and families [44]. Staff can educate and provide respectful, practical and immediate support to children and families for clean faces and hygiene practices to eliminate trachoma and reduce infectious disease in Indigenous children.

The multiple components of the health promotion intervention are shown in Table 1. This comprised of clinical education, community performances, football, Trachoma Story Kits, large safety mirrors, posters, television and radio adverts. The study found that despite the high levels of staff turnover and in a relatively short time, 70% of participants in all three settings reported they used or observed a health promotion intervention. This suggests trachoma resources and health promotion activities were associated with increased trachoma-related KAP as no other trachoma health promotion was functioning during the study period.

Trachoma programs in Australia and worldwide must bear in mind, that while increasing knowledge can occur in the short term, changing social norms, such as attitudes and behaviour, is a long-term process in any culture. Annual trachoma and clean face prevalence data from 2008–2015 in Table 3 shows a considerable reduction in trachoma prevalence over time. The study period of 2010–2012 is highlighted, during which trachoma prevalence reduced from 15% to 4%, possibly explained by widespread antibiotic treatment. The slight increase in trachoma prevalence after 2013 must be interpreted cautiously, but is thought to have been influenced by extended delays in program funding and activity. There was a marginal increase in clean face prevalence during the study period, which is not surprising as it should not be expected that meaningful and lasting behaviour change could occur within 1–2 years. From 2010 to 2015 however, a gradual but steady increase of clean face prevalence is clearer. Behaviour change takes time.

**Table 3:** The prevalence of trachoma and clean faces in children aged 5–9 years in screened communities in the NT.

Year	Trachoma Prevalence	Clean Face Prevalence
2015	4.8%	85%
2014	5.9%	86%
2013	5.0%	78%
2012	4.0%	75%
2011	7.0%	74%
2010	15.0%	74%
2009	15.0%	76%
2008	25.0%	73%

Period of Study August 2010 to July 2012.

Data Source: National Trachoma Surveillance and Reporting Unit, Australia.

In Australia, to support the F of the SAFE strategy, the health promotion priority was to increase trachoma awareness, provide culturally relevant health promotion approaches and create a workforce with the capacity to support changes in remote communities. To do so however, staff and community members require environmental improvements (E of SAFE). In many remote communities, there is a lack of properly installed and regularly maintained washing (bathroom) facilities in houses, schools, preschools, sporting areas and other community settings. Safe and functional bathroom facilities have sinks and showers with working taps and drains and include soap, paper towels and child height mirrors.

### Clinic

The greatest improvement in trachoma related KAP was observed in the clinic setting. The improvement in identifying trachoma was encouraging, but not critical as screening and treatment is the responsibility of visiting trachoma nurses with limited support by health clinics. Health clinics, though, have a vital role in educating children and families about trachoma and the risks of ocular and nasal secretions on children’s faces. Clinical skills to screen for trichiasis and to make timely referrals are also indispensable. Self-reported diagnostic skills for trichiasis increased from 51% to 74%, but should improve further. Clinic staff must actively and confidently promote clean faces with families when small children are present, clearly explaining the risk of transmission for trachoma and other infectious disease which is unacceptably high in remote Indigenous communities [45,46]. Health services should ensure that soap, washing facilities, tissues and large mirrors are easily accessible in clinics.

### Schools

Schools are highly involved in trachoma elimination programs. The trachoma and clean face prevalence data is gathered in annual (or six monthly) visits by nurses who undertake screening and treatment and health education. This study assessed the capacity of staff in schools as trachoma and hygiene health promotion rely solely on individual teachers and principals which can be fleeting due to constant staff turnover. The KAP of participants in schools did improve but they started with a lower baseline in most measures compared to the other groups and just 50% reported seeing a health promotion activity. Study participants from schools show significant improvement in understanding trachoma is ‘simple to treat’ and reported a capacity to ‘teach others about trachoma prevention’ both are good indicators of support for trachoma elimination programs. As many school settings in Australia have improved health equity through nutrition programs [25], it would make sense to incorporate hygiene practices and enhance student health and wellbeing in remote communities and ensure that soap, safe and functional washing facilities, tissues and large mirrors are accessible and maintained. The new Australian School Curriculum includes ‘face washing’ which offers an opportunity for the Departments of Education in each jurisdiction to operationalise clean faces and good hygiene practices in trachoma endemic regions.

### Community workplace

Participants from community work-setting showed positive changes in the proportion of reported trachoma elimination knowledge, attitudes and practices. The trend for higher levels of awareness is very encouraging as this group employs the largest proportion of community members and leaders. These include; government services and NGOs, Families as First Teachers, playgroups, preschools, family support organisations, community development programs, youth services and sport and recreation programs. These settings are important for families; they often incorporate local culture and language and offer supportive and practical activities for health and wellbeing programs including trachoma and hygiene health promotion. Community work setting staff were the group most engaged with the trachoma resources and almost 80% noted at least one of the initiatives. Further support for this wide-ranging setting in trachoma elimination activities is warranted and could lead to community control and local leadership for trachoma elimination.

## Strengths and limitations

Strengths of the study include having a wide geographical reach, covering three-quarters of the remote communities at risk of trachoma in the NT. The KAP survey provided high utility with difficult to reach participants in a program with very limited resources. Remote communities in Australia are especially difficult to survey due to research fatigue, high mobility of community members, language barriers and difficulty in accessing participants in busy, remote work places. Sufficient pre- and post-sampling rates were achieved by delivering surveys in person, following up when required by telephone and email and by the short questionnaire design. The program and study reach within the community was widespread with participants drawn from three key settings with daily contact and influence with community members and their children.

However, self-report KAP surveys have known weaknesses and several limitations must be considered when interpreting the results of this study. This survey relied on the participant’s introspection and interpretation of questions which may have resulted in over-reporting of KAP. There was also potential for response bias/courtesy bias where participants may have respond with answers they believe acceptable. The question “Are children’s dirty faces normal in your community?” is ambiguous and a weakness in the study. Every-day English was used in the survey to encourage participation by Indigenous community staff members. Although ‘normal’ is frequently used to mean *commonly observed* in colloquial language, the term could have been interpreted by some as either ‘common’ or ‘acceptable’. Nevertheless, either interpretation indicates a serious risk factor for trachoma. The question “I am able to teach others about trachoma prevention” should have been independently verified/assessed through supervision. Due to the turnover of staff, the program depended less on the retention of information by individual staff but in the implementation of an ongoing system to induct new staff and inculcate the information into the work setting. The resources and activities were a method of addressing the need for ongoing orientation and teaching about trachoma for new staff. Incorporating community-level evaluation was not possible due to resource limitations but the use of qualitative methods could have revealed important contextual and cultural factors and strengthened the study by validating results and reducing possible bias. The mobility of community and staff may have resulted in some participants being exposed to the health promotion intervention prior to baseline data being collected. It is anticipated that such exposure was limited due to the restrictions on mobility imposed in these communities by the wet season. While increased knowledge was noted and some changes to attitudes and practice, it cannot be assumed that it leads to a direct increase in health behaviour action. Equally, improved KAP among staff members may not positively influence or improve the health behaviours in trachoma endemic communities. It is recommended that further evaluation is planned to assess change in community level knowledge, attitude and practice.

## Conclusion

It is true that many health and social issues are more pressing than the elimination of trachoma in remote Indigenous communities, yet resolving them will take considerable time, resources and commitment by Indigenous and non-Indigenous Australians working across multiple sectors. Trachoma elimination does require resources, inter-sectoral commitment and working together, but it is more discrete than most other issues and can be addressed in a reasonably short time using the SAFE strategy. Importantly, improving safe and functional bathroom facilities and hygiene behaviours could reduce the unacceptably high burden of infectious disease.

The health promotion interventions in this study contributed to improved trachoma knowledge, attitudes and some practices of staff members in key work settings in remote communities. These results were found despite persistent staff turnover, which may indicate that some practices had been operationalised within the work settings rather than dependent on individual staff. The multiple approach, settings-based health promotion should be continued and community engagement and leadership increased, for sustainable, behaviour change for trachoma elimination. Longer term, the social determinants contributing directly to trachoma, including poorly functioning bathroom facilities and overcrowded housing, must be addressed at every level of government. These environmental improvements must be supported by health education for community-wide benefit and reduction in transmission of infection.

The findings of this study may provide some general lessons with wider relevance for trachoma elimination, yet it is recognised there is no ‘one-size-fits-all’ approach. Each region and each country has its unique culture, language, political settings, financial resources, administrative capacity and historical context. In Australia in the early stages of the trachoma elimination program, it was important to lay a foundation of support with culturally relevant health promotion and increased workforce skills and capacity. This study illustrates that one cannot assume clinic, school and community-support staff are well informed about trachoma and can guide the behaviour of communities without the support of tailored trachoma education, health promotion resources and strategies.

## Supporting information

S1 Checklist Strobe checklist. (PDF)
